# CXCL8 derived from tumor-associated macrophages and esophageal squamous cell carcinomas contributes to tumor progression by promoting migration and invasion of cancer cells

**DOI:** 10.18632/oncotarget.22526

**Published:** 2017-11-20

**Authors:** Masayoshi Hosono, Yu-Ichiro Koma, Nobuhisa Takase, Naoki Urakawa, Nobuhide Higashino, Kazuki Suemune, Himiko Kodaira, Mari Nishio, Manabu Shigeoka, Yoshihiro Kakeji, Hiroshi Yokozaki

**Affiliations:** ^1^ Division of Pathology, Department of Pathology, Kobe University Graduate School of Medicine, Chuo-ku, Kobe 650-0017, Japan; ^2^ Division of Gastro-intestinal Surgery, Department of Surgery, Kobe University Graduate School of Medicine, Chuo-ku, Kobe 650-0017, Japan

**Keywords:** CXCL8, esophageal squamous cell carcinoma, tumor-associated macrophage, tumor progression, tumor microenvironment

## Abstract

Tumor-associated macrophages (TAMs) are involved in tumor progression and poor prognosis in several malignancies. We previously demonstrated the interaction between high numbers of infiltrating TAMs and poor prognosis in esophageal squamous cell carcinomas (ESCCs). To investigate the significance of TAMs in ESCC, we conducted a cDNA microarray analysis of peripheral blood monocytes (PBMo)-derived macrophages and PBMo-derived macrophages stimulated with conditioned media of TE-series ESCC cell lines (TAM-like PBMo-derived macrophages). *C-X-C motif chemokine ligand 8* (*CXCL8*) was up-regulated in the TAM-like PBMo-derived macrophages. Here we confirmed a high expression level of CXCL8 in TAM-like PBMo-derived macrophages and the expression of CXCR1/2, known as CXCL8 receptors, in TE-series ESCC cell lines. Recombinant human CXCL8 induced the ESCC cell lines’ migration and invasion by the phosphorylation of Akt and Erk1/2. In indirect co-cultures, not only signal pathway inhibitors but also neutralizing antibodies against CXCL8, CXCR1 and CXCR2 suppressed these phenotypes induced by TAM-like PBMo-derived macrophages. Immunohistochemical analysis of 70 resected ESCC samples showed that high expression levels of CXCL8 in ESCC tissues were significantly associated with lymph node metastasis and poor prognosis. These results suggest that CXCL8 up-regulated in the microenvironment may contribute to ESCC progression by promoting cancer cells’ migration and invasion.

## INTRODUCTION

The tumor microenvironment has important roles in tumor progression [1]. Cancer stroma consists of non-cancer cells such as fibroblasts, macrophages, neutrophils, natural killer cells, dendritic cells, vascular endothelial cells and extracellular matrix. Macrophages are the main cellular component of the tumor microenvironment. From an oncologic viewpoint, macrophages have two different functions; i.e., as tumor suppressive (M1) or tumor supportive (M2) cells. M2 macrophages, which express high interleukin (IL)-10 and low IL-12, play roles in the wound healing process, allergies, and pro-tumoral activity. They display the specific receptors known as hemoglobin scavenger receptor (CD163), macrophage scavenger receptor I (CD204), and mannose receptor (CD206) [2, 3]. Tumor-associated macrophages (TAMs) polarize into the M2-like phenotype and are involved in tumor growth, invasion, angiogenesis and metastasis formation [4]. In several cancers, a high level of TAMs infiltrating into the tumor site is associated with poor prognosis [5–8].

In 2012, esophageal cancer was the eighth most common cancer and sixth leading cause of cancer-associated mortality worldwide, at an estimated 455,800 esophageal cancer cases and 400,200 cancer deaths [9]. In the Western world, esophageal adenocarcinoma is a common histological subtype of esophageal cancer. In contrast, in Asian countries including Japan, squamous cell carcinoma accounts for > 90% of esophageal cancer cases, but the number of esophageal adenocarcinomas is increasing in Asian countries [9, 10]. Not only alcohol and smoking but also genetic factors such as *aldehyde dehydrogenase 2* (*ALDH2*) and *alcohol dehydrogenase 1B* (*ADH1B*) have been identified as risks for esophageal squamous cell carcinoma (ESCC) [11, 12]. As ESCCs have a tendency to metastasize to lymph nodes in the early stage, ESCC patients tend to show poor prognoses.

Using immunohistochemistry, we previously demonstrated that a high number of CD204-positive TAMs infiltrating the tumor site are significantly associated with the histological grade, depth of tumor invasion, vessel invasion, lymph node metastasis, clinical stage, and poor prognosis in ESCC patients [13]. To explore the interaction between ESCCs and TAMs, we compared gene expression profiles between peripheral blood monocyte (PBMo)-derived macrophages and PBMo-derived macrophages stimulated with conditioned media of TE-series ESCC cell lines (TECM) (TAM-like PBMo-derived macrophages) by performing a cDNA microarray analysis [14]. Among up-regulated molecules, we previously demonstrated that GDF15 was associated with poor prognosis, promoting the migration of ESCC cells [14] and that NCAM accelerated the migration and survival of TAM-like PBMo-derived macrophages [15].

In the present study, we focused on one of the up-regulated genes in TAM-like PBMo-derived macrophages, *C-X-C motif chemokine ligand 8* (*CXCL8*). CXCL8 is also known as interleukin-8 (IL-8), and it activates receptors CXCR1 and CXCR2. CXCL8 was reported to be associated with tumor progression in breast cancer [16], colorectal cancer [17], non-small cell lung cancer [18], gastric cancer [19] and melanoma [20]. However, the interaction between CXCL8 derived from TAMs and ESCCs has not been established. Our present findings demonstrate the critical role and biological effect of CXCL8 derived from TAMs in the ESCC microenvironment.

## RESULTS

### Expression of CXCL8 in PBMo-derived macrophages and TAM-like PBMo-derived macrophages

**Figure 1:**
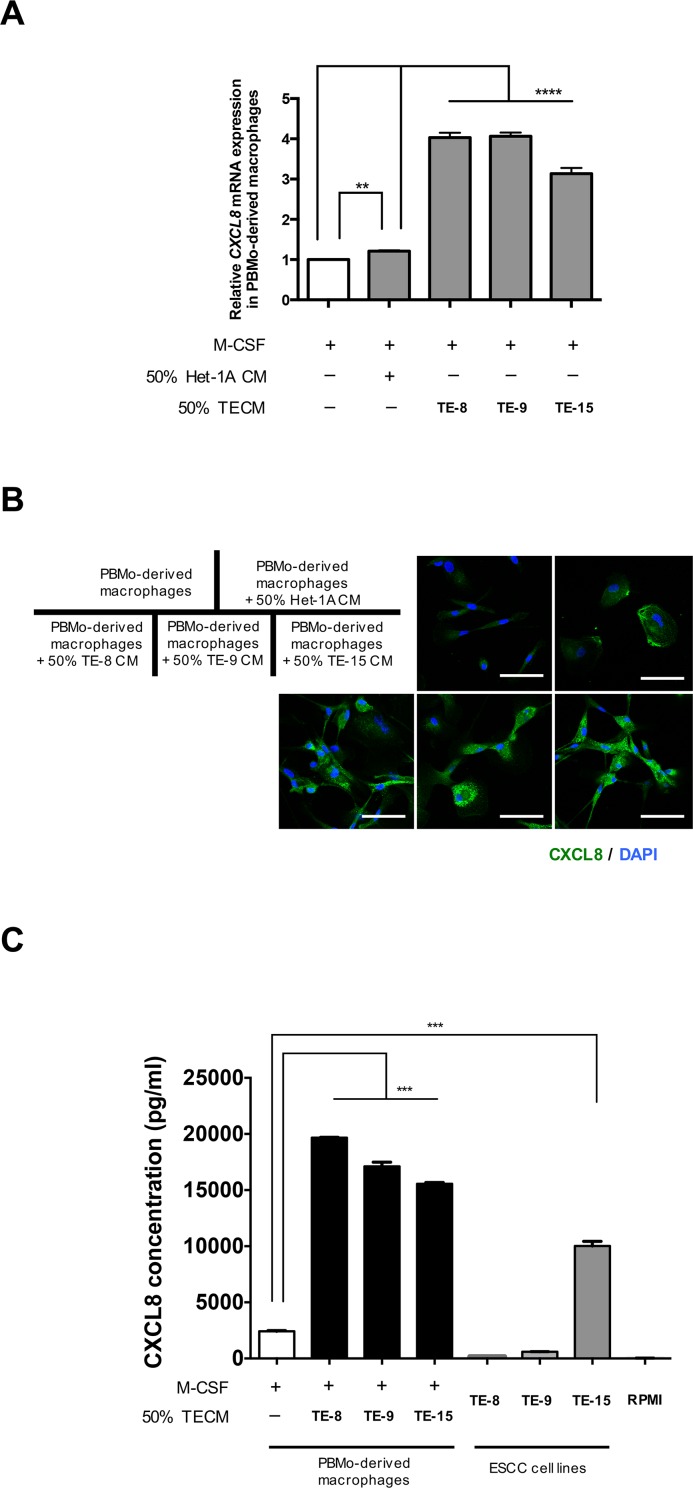
Induction of CXCL8 in PBMo-derived macrophages stimulated with TECM **(A)** The mRNA level of *CXCL8* in PBMo-derived macrophages stimulated with 50% TECM or 50% Het-1A CM was determined by quantitative RT-PCR. The data were normalized to *GAPDH* as an internal control. Data are mean ± SEM in triplicate. ^**^p < 0.01, ^****^p < 0.0001. **(B)** Expression of CXCL8 in PBMo-derived macrophages stimulated with TECM or Het-1A CM was confirmed by immunofluorescence using anti-CXCL8 antibody (green). Nuclei were stained with DAPI (blue). Magnification × 400. Scale bar, 50 μm. **(C)** Concentration of CXCL8 protein in conditioned medium of PBMo-derived macrophages stimulated with TECMs and ESCC cell lines. Protein levels were measured by ELISA. RPMI, negative control RPMI-1640 medium with serum. Data are mean ± SEM in triplicate. ^***^p < 0.001.

We first confirmed the expression level of CXCL8 in TAM-like PBMo-derived macrophages. The *CXCL8* mRNA expression was significantly up-regulated by TECM (TE-8, TE-9 and TE-15) compared to PBMo-derived macrophages and PBMo-derived macrophages stimulated by Het-1A CM (Figure 1A). The expression level of *CXCL8* was also significantly up-regulated by recombinant human (rh) IL- 4 compared to PBMo-derived macrophages (Supplementary Figure 1). Immunofluorescence demonstrated that CXCL8 was strongly expressed in the cytoplasm of TAM-like PBMo-derived macrophages (TE-8, TE-9 and TE-15) compared to the PBMo-derived macrophages and PBMo-derived macrophages stimulated by Het-1A CM (Figure 1B). The enzyme-linked immunosorbent assay showed that the concentration of secreted CXCL8 was significantly higher in the PBMo-derived macrophages stimulated with TE-8 CM, TE-9 CM and TE-15 CM (19664.8 ± 64.3, 17108.3 ± 601.8, and 15560.9 ± 179.4 pg/ml, respectively) than in the PBMo-derived macrophages (2423.8 ± 78.2 pg/ml) (Figure 1C). The concentration of CXCL8 secreted from TE-15 (10025.8 ± 711.6 pg/ml) was significantly higher than that in the PBMo-derived macrophages (Figure 1C). The concentration of CXCL8 derived from TE-8 and TE-9 (168.1 ± 13.0 and 596.4 ± 23.3 pg/ml) was lower than that in the PBMo-derived macrophages (Figure 1C).

### CXCL8 activated Akt and Erk1/2 via the CXCR1/2 of ESCC cells

**Figure 2:**
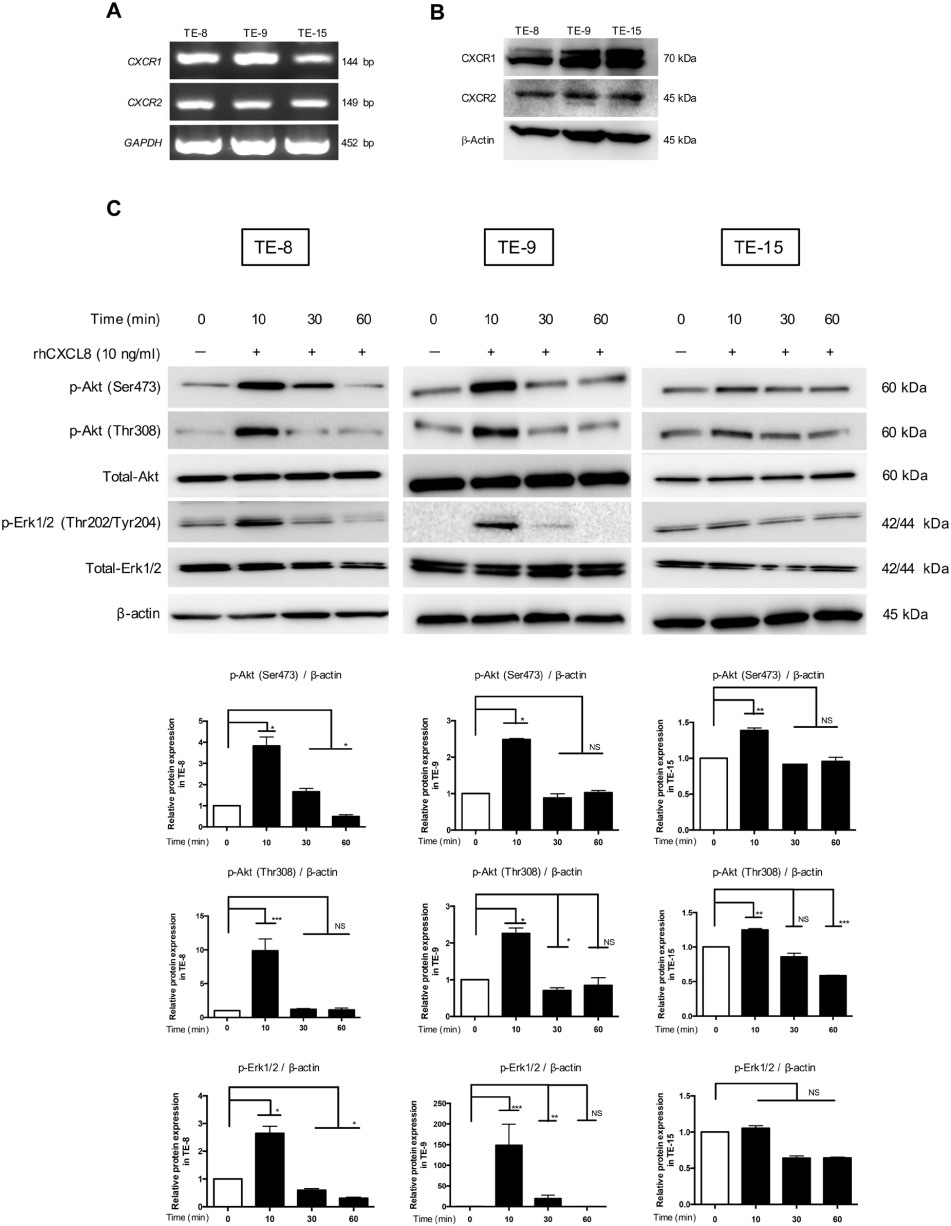
Akt and Erk1/2 were phosphorylated by CXCL8 through CXCR1 and CXCR2 in the ESCC cell lines **(A)** The mRNA levels of *CXCR1* and *CXCR2* in the ESCC cell lines were quantified by RT-PCR. **(B)** The protein level of CXCR1 and CXCR2 in the ESCC cell lines was confirmed by western blotting. Anti-CXCR1, CXCR2 and β-actin antibodies were used. **(C)** TE-8, TE-9 and TE-15 cells in serum-free conditions were treated with 10 ng/ml rhCXCL8 for 0, 10, 30 and 60 min. Western blotting was conducted with total protein extracted from ESCC cell lines using specific antibodies against Akt, p-Akt (Ser473), p-Akt (Thr308), Erk1/2, p-Erk1/2 (Thr202/Tyr204) and β-actin. Densitometric analysis of bands was performed with ImageJ (National Institutes of Health, Maryland, USA). The results are mean ± SEM. ^*^p < 0.05, ^**^p < 0.01, ^***^p < 0.001.

We confirmed the expressions of CXCR1 and CXCR2 (which are CXCL8 receptors) on the ESCC cell lines (TE-8, TE-9 and TE-15) by RT-PCR (Figure 2A) and western blotting (Figure 2B), respectively. To investigate the effect of CXCL8 on the post-receptor signaling of ESCC cells, we applied rhCXCL8 at 10 ng/ml to TE-8, TE-9 and TE-15 under serum-free conditions. We observed the phosphorylation of Akt (Ser473/Thr308) (the PI3K-Akt signal pathway) and Erk1/2 (the MEK-Erk1/2 signal pathway) after 10 min (Figure 2C).

### CXCL8 induced the migration and invasion of TE-8 and TE-9 cells

**Figure 3:**
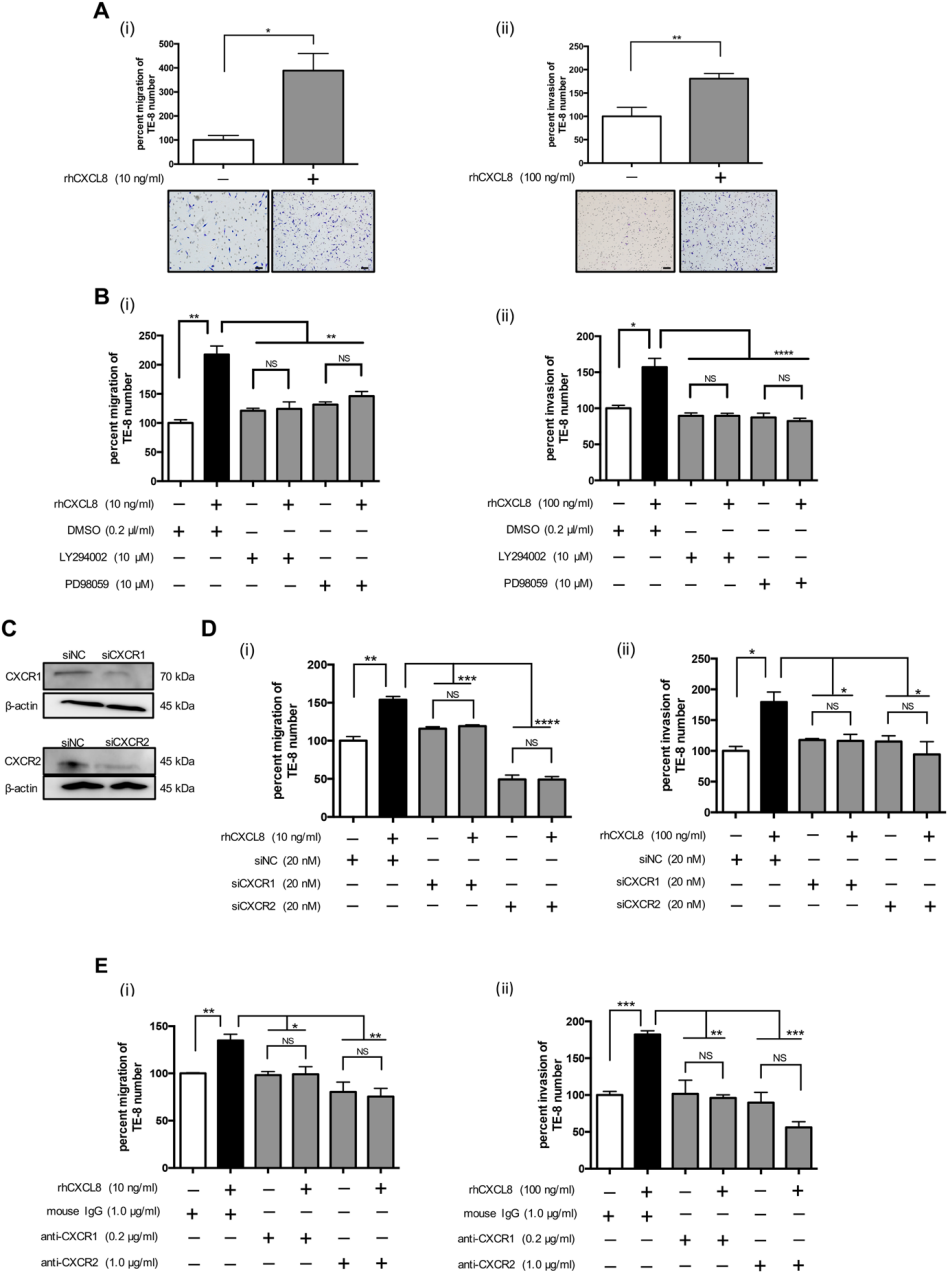
CXCL8 promoted the migration and invasion of the TE-8 cells **(A)** (i) For the transwell migration assay, TE-8 cells were plated on the transwell in serum-free RPMI-1640 at 5.0 × 10^5^ cells/well. rhCXCL8 was added in the upper chamber at 10 ng/ml. The cell inserts were set on 24-well plates in RPMI-1640 with 1% FBS for 24 h. The migrated cells on the underside of the membrane were stained by Diff-Quik and counted. The results are mean ± SEM. Scale bar, 100 μm. (ii) For the transwell invasion assay, TE-8 cells were seeded on a transwell coated with matrigel in serum-free RPMI-1640 at 5.0 × 10^5^ cells/well. Recombinant human CXCL8 was added in the transwell at 100 ng/ml. The cell inserts were set on 24-well plates in RPMI-1640 with 1% FBS for 48 h. The invaded cells on the underside of the membrane were stained by Diff-Quik and counted. **(B)** (i) TE-8 cells were plated on the upper chamber with or without rhCXCL8 at 10 ng/ml combined with the inhibitor against PI3K (LY294002, 10 μM) or MEK1/2 (PD98059, 10 μM). DMSO (0.2 μl/ml) was added to negative control. After 24 h, the migrated cells were counted. (ii) TE-8 cells were plated on the upper transwell coated with matrigel. rhCXCL8 was added in the transwell combined with LY294002 (10 μM) or PD98059 (10 μM). DMSO (0.2 μl/ml) was added to negative control. After 48 h, the invaded cells were counted. **(C)** TE-8 cells were transfected with 20 nM siRNA targeting *CXCR1* and *CXCR2*. siNC was transfected to TE-8 as negative control. Effective knockdown of CXCR1 and CXCR2 was confirmed by western blotting using antibodies against CXCR1 and CXCR2. **(D)** (i) *CXCR1*- or *CXCR2-*silenced TE-8 cells were plated on the upper transwell with rhCXCL8 at 10 ng/ml. After 24 h, the migrated cells were counted. (ii) *CXCR1*- or *CXCR2*-silenced TE-8 cells were plated on the upper transwell coated with matrigel. rhCXCL8 was added in the transwell at 100 ng/ml. After 48 h, the invaded cells were counted. **(E)** (i) TE-8 cells were plated on the upper transwell with rhCXCL8 at 10 ng/ml combined with the neutralizing antibody against CXCR1 (0.2 μg/ml) or CXCR2 (1.0 μg/ml). Mouse IgG was added to negative control. The concentrations of neutralizing antibodies were based on the manufacturer's instructions. (ii) TE-8 cells were plated on the upper transwell coated with matrigel. rhCXCL8 was added in the upper transwell at 100 ng/ml combined with the neutralizing antibody against CXCR1 (0.2 μg/ml) or CXCR2 (1.0 μg/ml). Mouse IgG was added to negative control. After 48 h, the invaded cells were counted. NS, not significant; ^*^p < 0.05, ^**^p < 0.01, ^***^p < 0.001, ^****^p < 0.0001.

First, we demonstrated rhCXCL8 did not promote the migration of TE-15 (expressing high level of CXCL8) (Supplementary Figure 2A) and neutralizing antibody against CXCL8 tended to suppress its migration (Supplementary Figure 2B). As we subsequently assessed the effect of CXCL8 derived from TAMs on the phenotype of the ESCC cell lines, we used TE-8 and TE-9 cells expressing low levels of CXCL8. We confirmed that rhCXCL8 had no effect on the proliferation or survival of TE cells (Supplementary Figure 3). We found that rhCXCL8 significantly accelerated the migration and invasion of TE-8 and TE-9 cells by performing a transwell migration assay and transwell invasion assay (Figure 3A (i)–(ii), Supplementary Figure 4A (i)–(ii)). LY294002, a PI3K inhibitor, and PD98059, a MEK1/2 inhibitor, suppressed the migration and invasion of TE-8 and TE-9 cells induced by rhCXCL8 (Figure 3B (i)–(ii), Supplementary Figure 4B (i)–(ii)).

We next suppressed the *CXCR1* and *CXCR2* of TE-8 and TE-9 cells by RNA interference. The silencing levels of *CXCR1* and *CXCR2* of TE-8 cells were confirmed by western blotting (Figure 3C). CXCL8 did not stimulate the migration and invasion of CXCR1- or CXCR2-silenced TE-8 and TE-9 cells (Figure 3D (i)–(ii), Supplementary Figure 4C (i)–(ii)). The neutralizing antibodies against CXCR1 or CXCR2 reduced the migration and invasion of TE-8 and TE-9 cells induced by rhCXCL8 (Figure 3E (i)–(ii), Supplementary Figure 4D (i)–(ii)).

### Inhibitors against PI3K and MEK1/2 and neutralizing antibodies against CXCL8, CXCR1, and CXCR2 suppressed the migration and invasion of TE-8 and TE-9 cells induced by TAM-like PBMo-derived macrophages

**Figure 4:**
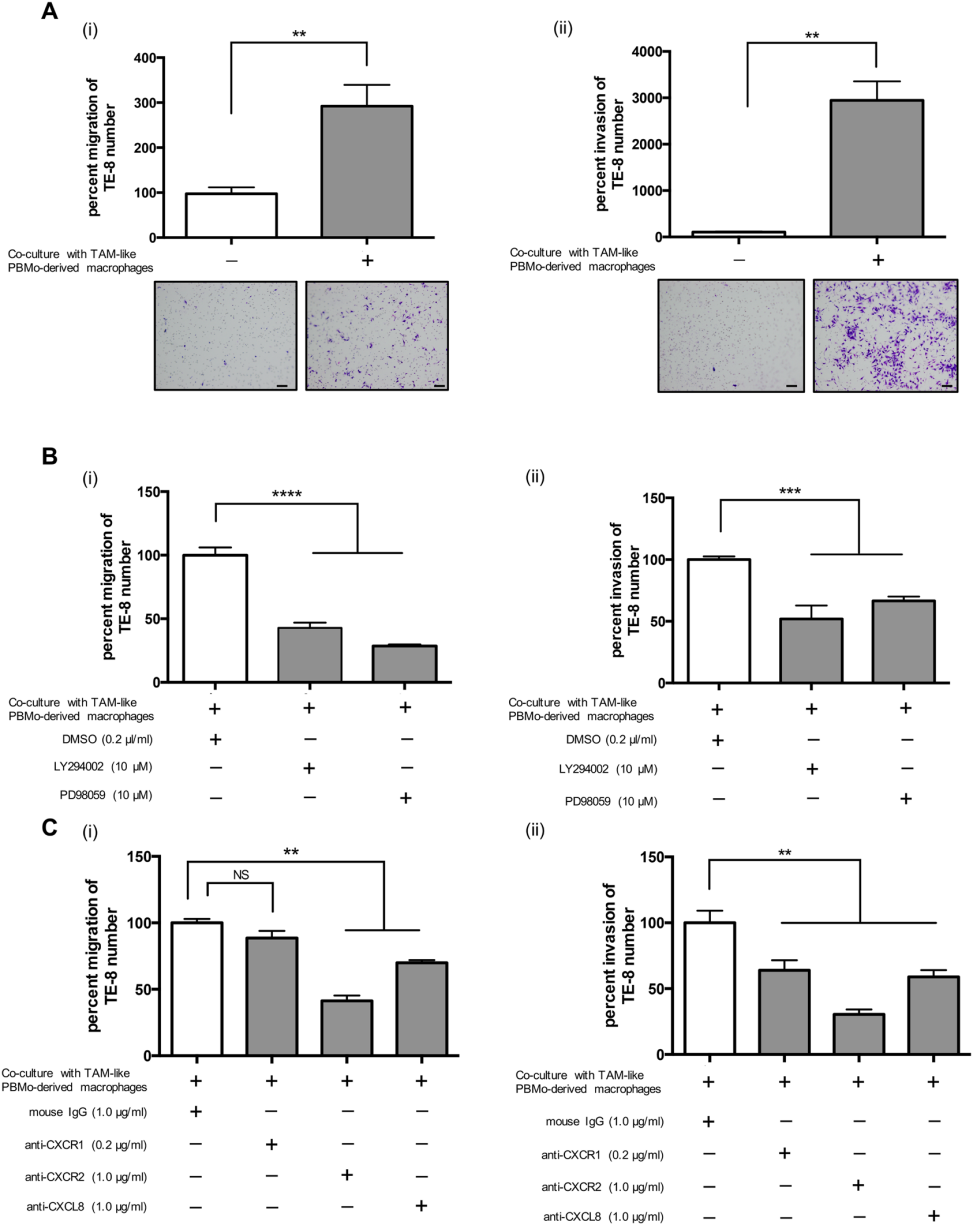
TAM-like PBMo-derived macrophages induced the migration and invasion of TE-8 cells by activating PI3K and MEK1/2 signals through CXCL8 and CXCR1/2 interaction **(A)** PBMos (1.0 × 10^5^ cells/well) were seeded on the lower chamber of 24-well plates with M-CSF (25 ng/ml) for 6 days to induce PBMo-derived macrophages, then incubated with 50% TE-8 CM to induce TAM-like PBMo-derived macrophages. After 2 days, the media were replaced with serum-free media. (i) TE-8 cells were plated on the upper transwell at 5.0 × 10^5^ cells/well and set on the plate. After 24 h, the migrated cells were counted. (ii) TE-8 cells were plated on the upper transwell coated with matrigel at 5.0 × 10^5^ cells/well and set on the plate. After 48 h, the invaded cells were counted. Scale bar, 100 μm. **(B)** (i) TE-8 cells were plated on the upper transwell with the inhibitor against PI3K (LY294002, 10 nM) or MEK1/2 (PD98059, 10 nM). DMSO (0.2 μl/ml) was added to negative control. After 24 h, the migrated cells were counted. (ii) TE-8 cells were plated on the upper matrigel-coated transwell with LY294002 (10 nM) or PD98059 (10 nM). DMSO (0.2 μl/ml) was added to negative control. After 48 h, the invaded cells were counted. **(C)** (i) TE-8 cells were plated on the upper transwell with the neutralizing antibodies against CXCR1 (0.2 μg/ml), CXCR2 (1.0 μg/ml) or CXCL8 (1.0 μg/ml). Mouse IgG was added to negative control. After 24 h, the migrated cells were counted. The concentrations of neutralizing antibody were based on manufacturer's instructions. (ii) TE-8 cells were plated on the upper matrigel-coated transwell with the neutralizing antibodies against CXCR1 (0.2 μg/ml), CXCR2 (1.0 μg/ml) or CXCL8 (1.0 μg/ml). Mouse IgG was added to negative control. After 48 h, the invaded cells were counted. The results are mean ± SEM. ^*^p < 0.05, ^**^p < 0.01, ^***^p < 0.001, ^****^p < 0.0001.

We investigated whether TAM-like PBMo-derived macrophages promoted the migration and invasion of ESCC cells. Our results demonstrated that TAM-like PBMo-derived macrophages significantly induced the migration and invasion of TE-8 and TE-9 cells (Figure 4A (i)–(ii), Supplementary Figure 5A (i)–(ii)). LY294002 or PD98059 inhibited the migration and invasion of TE-8 and TE-9 cells induced by TAM-like PBMo-derived macrophages (Figure 4B (i)–(ii), Supplementary Figure 5B (i)–(ii)). The neutralizing antibodies against CXCL8 and CXCR2 significantly suppressed the migration of TE-8 cells induced by TAM-like PBMo-derived macrophages, and the neutralizing antibodies against CXCR1 tended to reduce the migration of TE-8 cells (Figure 4C (i)). These neutralizing antibodies significantly inhibited the invasion of TE-8 cells induced by TAM-like PBMo-derived macrophages (Figure 4C (ii)). The neutralizing antibodies against CXCR1, CXCR2 and CXCL8 also suppressed migration and invasion of TE-9 cells induced by TAM-like PBMo-derived macrophages (Supplementary Figure 5C (i)–(ii)).

### Not only TAMs but also cancer cells expressed CXCL8 in human ESCC tissue

**Figure 5:**
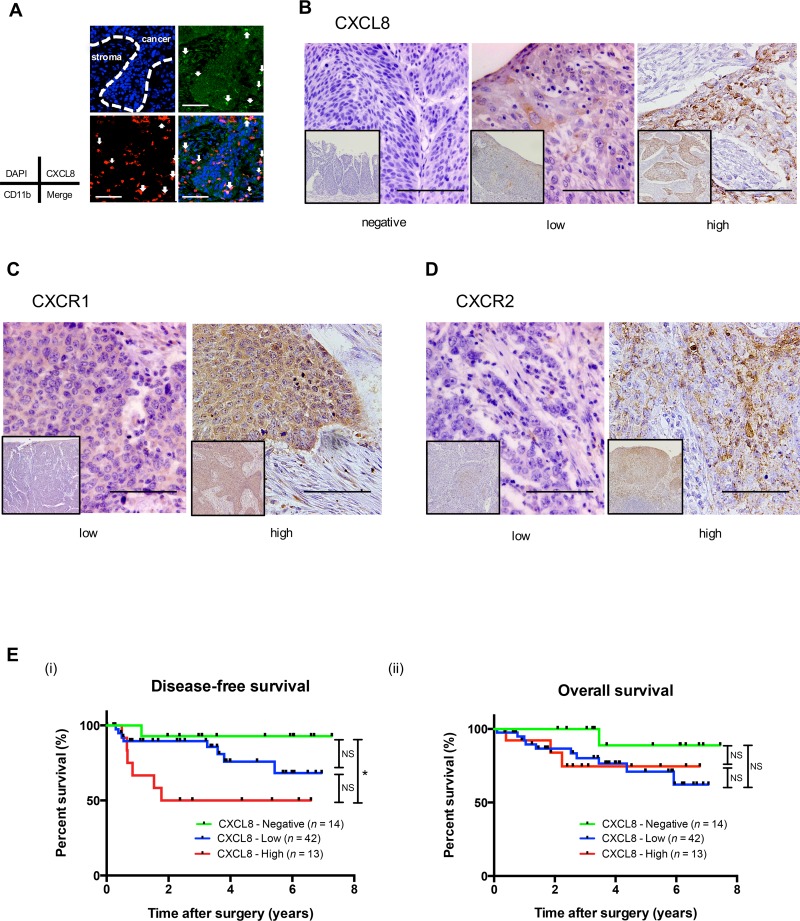
A high expression of CXCL8 in human ESCC tissues is associated with the poor prognosis of ESCC patients **(A)** A double immunofluorescence analysis was performed using anti-CXCL8 (green) and anti-CD11b (red) antibodies on human ESCC tissue. Nuclei were stained with DAPI (blue). Not only CD11b-positive macrophages but also the cancer nest was stained by CXCL8 in human ESCC tissue. Scale bar, 100 μm. **(B)** Immunohistochemical staining for CXCL8 in human ESCC tissue. Typical images of CXCL8 are shown: negative (*left*), low-intensity (*center*) and high-intensity (*right)* compared to the corresponding normal esophageal epithelia. Scale bar, 100 μm. **(C)** Immunohistochemical staining for CXCR1 in human ESCC tissue. Typical images are shown: low-intensity (*left*) and high-intensity (*right*) compared to the corresponding normal esophageal epithelia. Scale bar, 100 μm. **(D)** Immunohistochemical staining for CXCR2 in human ESCC tissue. Typical images are shown: low-intensity (*left*) and high-intensity (*right*) compared to the corresponding normal esophageal epithelia. Scale bar, 100 μm. **(E)** Kaplan-Meier analysis of ESCC patients divided into three groups according to their expression of CXCL8: CXCL8-Negative group (n = 14), CXCL8-Low group (n = 43) and CXCL8-High group (n = 13). The log-rank test was performed to determine significance. ^*^p < 0.05.

We examined the expression of CXCL8 in human ESCC tissue samples. Immunofluorescence demonstrated that a large fraction of the CXCL8-positive spindle cells co-expressed with the macrophage marker CD11b in the tumor nests and stroma of human ESCCs (Figure 5A, Supplementary Figure 6A). We observed CXCL8 expression in normal squamous epithelia and tumor nests, particularly in the invasive area, by immunohistochemistry. We observed various levels of CXCL8 immunoreactivities in cancer nests (negative, low and high) using corresponding normal squamous epithelia as a positive control (Figure 5B, Supplementary Figure 6B).

In non-neoplastic esophageal tissues, CXCR1 and CXCR2 were expressed in normal squamous epithelia and smooth muscle. In the tumor nests of human ESCC tissues, various levels of CXCR1 or CXCR2 were observed: low and high compared to the normal squamous epithelia as a positive control (Figure 5C and 5D).

### The expression levels of CXCL8 were closely correlated with clinicopathological factors and the prognosis of the ESCC patients

We investigated whether the expression of CXCL8 had any significant association with clinicopathological factors of ESCC patients. We found that a high expression of CXCL8 was closely correlated with lymph node metastasis (*p* = 0.039), a high number of infiltrating CD204-positive macrophages (*p* = 0.043), and a high expression level of CXCR2 (*p* = 0.043) (Table 1).

**Table 1:** Expression level of CXCL8 in ESCC and their correlation with clinicopathological data and infiltrating macrophages phenotypes

	Number of cases	Expression of CXCL8^a^			*p*-value
		Negative (*n* = 14)	Low (*n* = 43)	High (*n* = 13)	
Age					
< 65	33	8	18	7	0.528
≥ 65	37	6	25	6	
Histological grade^b^					
HGIEN + WDSCC	16	4	9	3	0.986
MDSCC + PDSCC	54	10	34	10	
Depth of tumor invasion^b^					
T1	49	12	31	6	0.072
T2 + T3	21	2	12	7	
Lymphatic vessel invasion^b^					
Negative	37	9	23	5	0.402
Positive	33	5	20	8	
Blood vessel invasion^b^					
Negative	43	9	28	6	0.447
Positive	27	5	15	7	
Lymph node metastasis^b^					
Negative	43	9	30	4	0.039^*^
Positive	27	5	13	9	
Stage^c^					
0 + I	38	7	27	4	0.276
II + III + IV	32	7	16	9	
CD68 positive cells^d^					
Low	35	10	21	4	0.104
High	35	4	22	9	
CD163 positive cells^d^					
Low	35	9	22	4	0.213
High	35	5	21	9	
CD204 positive cells^d^					
Low	34	10	21	3	0.043^*^
High	36	4	22	10	
CXCR1					
Low	41	9	24	8	0.831
High	29	5	19	5	
CXCR2					
Low	37	11	22	4	0.043^*^
High	33	3	21	9	

Data were analyzed by χ^2^-test. *p* < 0.05 was considered statistically significant; ^*^p < 0.05

^a^Expression of CXCL8 in invasive area of ESCC tissue was divided into three groups according compared with the corresponding normal esophageal epithelia; Negative, Low and High.

^b^According to the Japanese Classification of Esophageal Cancer. HGIEN, high-grade intraepithelial neoplasia; WDSCC, well-differentiated squamous cell carcinoma; MDSCC, moderately differentiated squamous cell carcinoma; PDSCC, poorly differentiated squamous cell carcinoma. T1a, tumor invades mucosa; T1b, tumor invades submucosa; T2, tumor invades muscularis propria; T3, tumor invades adventitia.

^c^According to the TNM classification by UICC.

^d^The median values of CD68 positive, CD163 positive or CD204 positive macrophage numbers in cancer nests and stroma within the areas were used to divide the patients into low- and high-groups.

We next performed a prognostic study of 69 of the 70 ESCC patients (excluding the single patient who could not be followed). The disease-free survival (DFS) of the patients with a high expression of CXCL8 was significantly shorter compared to that of the patients with a negative or low expression of CXCL8 by log-rank test (the percent DFS after 2 years of the CXCL8 high, low, and negative groups were 50%, 89.5%, and 92.85%, respectively, high vs. low vs. negative, *p* = 0.0265; high vs. low, *p =* 0.055; high vs. negative, *p =* 0.013) (Figure 5E (i)).

The overall survival of the patients was not significantly different among the negative-, low- and high-expression CXCL8 groups (*p =* 0.25) (Figure 5E (ii)). A significant independent impact of CXCL8 on the DFS rate was not revealed by the multivariate analysis in ESCC (Table 2). The expressions of CXCR1 or CXCR2 were not related to DFS or overall survival by log-rank test (Supplementary Figure 7).

**Table 2:** Relationship between clinicopathological features of human squamous cell carcinoma and disease-free survival

	Univariate analysis			Multivariate analysis		
	Number	Median survival (years)	*p*-value	HR	95% CI	*p*-value
Age						
< 65	33	4.93	0.212			
≥ 65	36	6.15				
Histological grade^a^						
HGIEN + WDSCC	16	5.83	0.551			
MDSCC + PDSCC	53	5.65				
Depth of tumor invasion^a^						
T1	49	6.53	< 0.001^***^	7.26	1.035–51.00	0.046^*^
T2 + T3	20	3.27				
Lymphatic vessel invasion^a^						
Negative	37	6.43	0.002^**^	1.19	0.219–6.463	0.841
Positive	32	55.3				
Blood vessel invasion^a^						
Negative	43	5.84	0.181			
Positive	26	5.11				
Lymph node metastasis^a^						
Negative	43	6.48	< 0.001^***^	0.57	0.046–7.155	0.666
Positive	26	4.16				
Stage^b^						
0 + I	38	6.62	< 0.001^***^	8.55	1.035–51.002	0.185
II + III + IV	31	4.34				
CXCL8						
Negative	14	7.01	0.026^*^	1.90	10.82–4.374	0.133
Low	42	5.68				
High	13	3.80				
CXCR1						
Low	40	5.56	0.524			
High	29	5.58				
CXCR2						
Low	38	5.54	0.46			
High	31	5.62				
CD68 positive cells^c^						
Low	35	5.96	0.034^*^	3.85	0.511–29.07	0.191
High	34	5.02				
CD163 positive cells^c^						
Low	35	6.0	0.134			
High	34	5.21				
CD204 positive cells^c^						
Low	34	6.2	0.025^*^	0.196	0.22–1.786	0.148
High	35	5.0				

Disease free survival was estimated by Kaplan-Meier method compared by log-rank test. *p* < 0.05 was considered statistically significant; ^*^p < 0.05; ^**^p < 0.01; ^***^p < 0.001.

^a^According to the Japanese Classification of Esophageal Cancer. HGIEN, high-grade intraepithelial neoplasia; WDSCC, well-differentiated squamous cell carcinoma; MDSCC, moderately differentiated squamous cell carcinoma; PDSCC, poorly differentiated squamous cell carcinoma. T1a, tumor invades mucosa; T1b, tumor invades submucosa; T2, tumor invades muscularis propria; T3, tumor invades adventitia.

^b^According to the TNM classification by UICC.

^c^The median values of CD68 positive, CD163 positive or CD204 positive macrophage numbers in cancer nests and stroma within the areas were used to divide the patients into low- and high-groups.

## DISCUSSION

CXCL8, also known as IL-8, belongs to the C-X-C motif chemokine family. The *CXCL8* gene encodes for a protein of 99 amino acids, and it is processed to active proteins of either 77 amino acids in nonimmune cells or 72 amino acids in immune cells. CXCR1 and CXCR2, G protein-coupled receptors, are known as CXCL8 receptors and are characterized by seven-transmembrane-spanning regions. CXCR1 is a receptor for CXCL6 and CXCL8, whereas CXCR2 is a receptor for not only CXCL8 but also CXCL1, CXCL2, CXCL3, CXCL5, CXCL6 and CXCL7 [21, 22]. Previous research demonstrated that CXCL8 binds to CXCR1 or CXCR2 and activates the PI3K-Akt and MEK1/2-Erk1/2 signaling pathways [23].

Recent studies have revealed that CXCL8 is up-regulated and involved in the tumor progression of various human malignancies, including prostate cancer [24], breast cancer [25], gastric cancer [26], non-small lung cancer [27], colon cancer [28] and melanoma [29]. In human ESCCs, it was shown that a high concentration of CXCL8 in the serum or high levels of tissue expression in human ESCCs were associated with distant metastasis and poor prognosis [30]. However, no study has investigated the biological mechanism of a CXCL8-CXCR1/CXCR2 axis with the evaluation of the expression level of CXCL8 in TAMs that have invaded ESCC tissue. In the present study, we confirmed the expression level of CXCL8 in PBMo-derived macrophages, TAM-like PBMo-derived macrophages and ESCC cell lines, and the expression of CXCR1 and CXCR2 on the ESCC cell lines. Furudate *et al.* reported PBMo-derived macrophages treated with IL-4 expressed high level of various chemokines, including CXCL8, compared with PBMo-derived macrophages [31]. We also demonstrated that rhIL-4 up-regulated the expression level of *CXCL8* in PBMo-derived macrophages (Supplementary Figure 1). We then observed that rhCXCL8 induced the phosphorylation of Akt and Erk1/2 in human ESCC cells lines, which was similar to the results of previous studies of other cancer cell lines.

It has been revealed that CXCL8 promoted cell migration and/or invasion in human gastric cancer [32], colon cancer [33], breast cancer [34], hepatocellular carcinoma [35] and prostate cancer *in vitro* [36]. CXCL8 has also been reported to promote the proliferation of human colon cancer [37], prostate cancer [38] and breast cancer cells [34]. In the present study, we demonstrated for the first time that rhCXCL8 significantly induced the migration and invasion of human ESCC cell lines. rhCXCL8 had no effect on the proliferation of the human ESCC cells. As was reported regarding a papillary thyroid carcinoma cell line [39], CXCL8 might have different functions depending on the origin and histological types of cancer cells.

Earlier studies indicate that CXCL8 derived from TAMs is associated with tumor progression. Using a cytokine array analysis, Fang *et al*. [39] reported that the CXCL8 expression of TAMs which were isolated from human thyroid papillary cancer tissue was up-regulated in comparison with that of peripheral blood monocytes from healthy donors. They observed that rhCXCL8 promoted the invasion of papillary thyroid carcinoma cell lines, K1, TPC-1 and BCPAP *in vitro*. They also reported that BCPAP injected into tail vein of rhCXCL8-treated mice tended to metastasize to lung compared to the control mice [39].

Cao *et al*. demonstrated the CXCL8 derived from TAMs stimulated by leptin promoted the migration and invasion of human breast cancer cell lines *in vitro* and promoted tumor growth *in vivo* [40]. Tong *et al*. showed that CXCL8 from TAMs promoted the cell invasion of endometrial cancer through suppressed ERα expression, which was related to poor survival [41]. In the present study, we demonstrated that CXCL8 derived from TAM-like PBMo-derived macrophages induced cell migration and invasion by indirect co-culture with ESCC cell lines, and these were significantly suppressed by neutralizing antibodies against CXCR1, CXCR2 or CXCL8. These observations indicate that CXCL8 derived from TAMs has important roles in the migration and invasion of ESCC cells.

CXCR2 is known as multiple C-X-C motif chemokine receptor. Wang *et al*. showed that CXCL1, one of the ligands to CXCR2, is involved in the migration and invasion of gastric cancer cells *in vitro* [42]. Using immunohistochemistry, they also showed that the expression level of CXCL1 in human gastric cancer tissues was associated with lymph node metastasis and TNM classification. Zhao *et al*. reported that the CXCL5-CXCR2 axis promoted the migration of human colorectal cancer cells *in vitro* and increased liver metastasis from the injected tumor of mice spleen *in vivo* [43]. Neutralizing antibody against CXCR2 demonstrated a stronger suppressive effect on the phenotypes of ESCC cell lines induced by TAM-like PBMo-derived macrophages compare to the antibodies against CXCR1 and CXCL8. Targeting CXCR2 to suppress the migration and invasion of ESCC cells by inhibiting the function of not only CXCL8 but also other C-X-C motif chemokines derived from TAMs may be a rather effective therapy for ESCC.

Using immunohistochemistry, Ogura *et al*. showed that high expression levels of CXCL8 and CXCR2 in human ESCC tissue were associated with poor prognosis [44]. They classified ESCC tissues into two groups, negative and positive, based on the product of the intensity of immunostaining and the percentage of positive tumor cells expressing CXCL8 and CXCR2. In the present study, we classified the expression level of CXCL8 into three groups: negative, low, and high. As low-dose CXCL8 was reported to induce the migration and invasion of human gastric cancer cell lines [45], we suspected that even a low expression level of CXCL8 would be associated with tumor progression. Our investigation revealed that a high expression level of CXCL8 by ESCC tissue was significantly associated with DFS, lymph node metastasis, a high expression level of CXCR2, and the invasion of high numbers of CD204-positive macrophages. Our findings indicated that CXCL8 derived from not only TAMs but also ESCC cells contributed to the progression of ESCC. In this study, although the combination of a high expression level of CXCL8 and that of CXCR2 was not significantly associated with poor prognosis, the CXCL8 expression level was significantly related to that of CXCR2. Using immunohistochemistry in human ESCC, Sui *et al*. reported that a high expression of CXCR2 was correlated with poor survival [46]. In our study, the expression levels of both CXCR1 and CXCR2 in cancer nests were not related to prognosis. Notably, this study had a limited number of patients for the evaluation of the interaction between the expression levels of receptors and prognosis.

**Figure 6:**
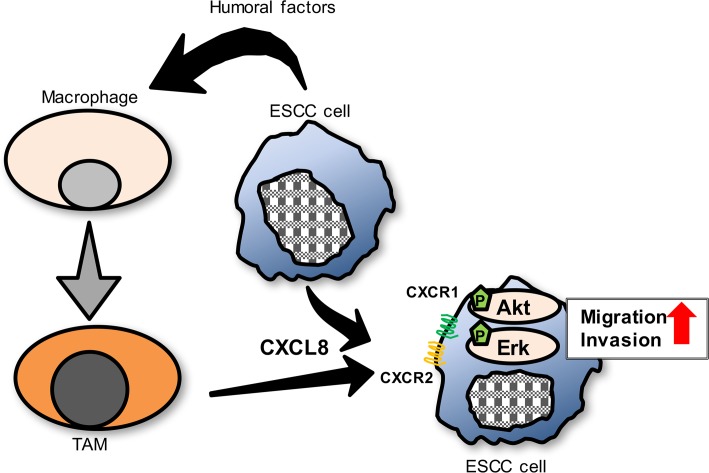
A proposed model of the interaction between ESCC cells and TAMs through CXCL8 in tumor microenvironment Many humoral factors, including IL-4, induced macrophages into TAMs. Then, expression level of CXCL8 was up-regulated in TAMs. CXCL8 secreted from TAMs, as well as from ESCC cells, contributed ESCC cells' migration and invasion by activating Akt and Erk1/2 signals through CXCR1/CXCR2 of ESCC cells.

In conclusion, we demonstrated that CXCL8 derived from TAM-like PBMo-derived macrophages promoted the migration and invasion of ESCC cell lines *via* the phosphorylation of Akt and Erk1/2 through CXCR1 and CXCR2 *in vitro*. A high expression level of CXCL8 in TAMs and ESCC cancer cells was strongly associated with poor prognosis through lymph node metastasis. Targeting the CXCL8-CXCR1/CXCR2 axis in human ESCC tissues might be an effective new therapy (Figure 6).

## MATERIALS AND METHODS

### Cell line and cell culture

Three ESCC cell lines (TE-8, TE-9 and TE-15) were obtained from the RIKEN BioResource Center (Tsukuba, Japan) [47]. Het-1A is a normal human squamous esophageal cell line transfected non-tumorigenic SV40-T. We purchased it from American Type Culture Collection^®^ (Manassas, VA, USA) [48]. The individuality of the TE series ESCC cell lines was confirmed by a short tandem repeat (STR) analysis at RIKEN and at the Cell Resource Center for Biomedical Research, Institute of Development, Aging and Cancer, Tohoku University in 2009 and 2010 (Sendai, Japan). All TE cells were confirmed to be mycoplasma-negative by a Venor^®^ Gem Classic Mycoplasma Detection kit (Minerva Biolabs, Berlin, Germany). Het-1A was confirmed not to have human pathogenic virus using PCR-based assay for HIV, HepB, HPV, EBV and CMV. We maintained the ESCC cell lines in RPMI-1640 (Wako, Osaka, Japan) with 10% fetal bovine serum (FBS) (Sigma-Aldrich, St Louis, MO, USA) and 1% antibiotic-antimycotic (Invitrogen, Carlsbad, CA). Het-1A cell line was cultured in BEGM™ (Lonza, Walkersvile, MD, USA). The conditioned medium of TE series (TECM) and Het-1A CM was prepared by plating 5 × 10^6^ tumor cells in 10 ml of complete medium in 100-mm dishes for 24 h, and the medium was then changed to complete DMEM (Wako) with 10% human AB serum (Lonza, Walkersville, MD). After 2 days, the supernatants were harvested, centrifuged, and stored in aliquots at − 80°C.

### Macrophage culture

Peripheral blood mononuclear cells (PBMCs) were obtained from healthy volunteer donors after informed consent was obtained. CD14^+^ peripheral blood monocytes (PBMos) were purified from PBMCs by positive selection using the auto MACS^®^ Pro Separator (Miltentyi Biotec, Bergisch Gladbach, Germany). PBMos were cultured with macrophage-colony stimulating factor (M-CSF) (25 ng/ml; R&D Systems, Minneapolis, MN) for 6 days to induce macrophages, then cultured for 2 days added 50% TECMs to induce TAM-like polarization.

### Tissue samples

A total of 70 human ESCC tissue samples excised from 2005 to 2010 at Kobe University Hospital (Kobe, Japan) were used in this study. Informed consent for the use of tissue samples was obtained from all patients, and the study was approved by the Kobe University Institutional Review Board. We analyzed the histological and clinicopathological information using the Japanese Classification of Esophageal Cancer proposed by the Japan Esophageal Society and the TNM classification of the Union for International Cancer Control.

### Immunofluorescence

TE cells were seeded onto coverslips overnight and fixed with 4% paraformaldehyde phosphate buffer solution (Wako) and incubated with rabbit monoclonal antibody against CD11b (1:100, #ab52478, Abcam, Cambridge, UK), mouse monoclonal antibody against CXCL8 (1:20, #ab18672, Abcam), rabbit polyclonal antibody against CXCR1 (1:50, #sc-988, Santa Cruz Biotechnology, Dallas, TX) and CXCR2 (1:50, #sc-682, Santa Cruz) at 4°C overnight. Alexa Fluor^®^ 488-conjugated donkey anti-mouse secondary antibody (Jackson ImmunoResearch Laboratories, West Grove, PA), Cy3-conjugated donkey anti-rabbit IgG secondary antibody (Jackson ImmunoResearch Laboratories) and DyLight 488-conjugated goat anti-mouse secondary antibody (Vector Laboratories, Burlingame, CA) were incubated at room temperature for 1 h. The nuclei were stained with DAPI (Wako). Images were taken with a Zeiss LSM 700 laser-scanning microscope and analyzed using the LSM software ZEN 2009 (Carl Zeiss, Oberkochen, Germany).

### Reverse transcription-PCR (RT-PCR) and quantitative RT-PCR (qRT-PCR)

Total RNA was extracted from cultured cells using the RNeasy Mini Kit (Qiagen, Hilden, Germany). RT-PCR amplifications of *CXCL8*, *CXCR1*, *CXCR2* and the internal control gene *GAPDH* were performed. PCR products were subjected to electrophoresis in a 2% agarose gel. The qRT-PCR amplifications of *CXCL8* and the internal control gene *GAPDH* were performed using the ABI StepOne Real-time PCR system (Applied Biosystems, Foster City, CA). The threshold cycle (Ct) values were determined by plotting the observed fluorescence against the cycle number. Ct values were analyzed using the comparative threshold cycle method and normalized to those of *GAPDH*.

The relative gene expression levels were estimated using following formula: relative expression = 2 – (Ct [target gene] – Ct [GAPDH]). The primers were designed as follows: *GAPDH*, 5′-ACC ACA GTC CAT GCC ATC AC-3′ / 5′-TCC ACC ACC CTG TTG CTG TA-3′; *CXCL8*, 5′-AAA CCA CCG GAA GGA ACC AT-3′ / 5′-CCT TCA CAC AGA GCT GCA GAA A-3′; *CXCR1*, 5′-CTG CAG CTC CTA CTG TTG G-3′ / 5′-TTC ATC TGC AGC TGG CAT G-3′; *CXCR2,* 5′-GGT TGC CAA GCC TTG TCT GA-3′ / 5′-AGG GAG TTC ACA TGC GCC T-3′

### Western blotting

Cells were lysed on ice with a cell lysis buffer (50 mM Tris-HCl pH 7.5, 125 mM NaCl, 0.1%Triton X-100 and 5 mM EDTA) or NP40 cell lysis buffer (Thermo Fisher Scientific, Waltham, MA) containing both 1% protease inhibitor and 1% phosphatase inhibitor cocktail (Sigma-Aldrich). The resulting lysates were separated on 5–20% sodium dodecyl sulfate polyacrylamide gels, transferred to a membrane with iBlot Gel Transfer Stack (Invitrogen). The membrane was blocked with 5% skim milk and then incubated with primary and secondary antibodies. The protein bands were detected with ImmunoStar Reagents (Wako).

The primary antibodies were as follows. Rabbit antibody against CXCR1 (1:50, #sc-988, Santa Cruz), rabbit antibody against CXCR2 (1:50; #sc-682, Santa Cruz), and the following (all from Cell Signaling Technology, Beverly, MA): rabbit antibody against phosphorylated Akt (Ser473, 1:500, #4060), rabbit antibody against phosphorylated Akt (Thr308, 1:500, #2965), rabbit antibody against non-phosphorylated Akt (1:1000, #9272), rabbit antibody against phosphorylated Erk1/2 (Thr202/Tyr204, 1:500, #9101), rabbit antibody against non-phosphorylated Erk1/2 (1:500, #9102), rabbit antibody against β-actin (1:1000, #4970).

The following secondary antibodies were both from GE Healthcare Life Sciences, Little Chalfont, UK: horseradish peroxidase (HRP)-linked sheep anti-mouse IgG (NA931V, NA931) and HRP-linked donkey anti-rabbit IgG (NA934V).

### Cell growth assay and survival assay

Cells were seeded on 96-well plates at 1.0 × 10^4^ per well with serum-free RPMI-1640 or seeded at 5.0 × 10^3^ per well with 1% FBS, followed by incubation at 37°C. The cells were then treated with 0, 1, 10 or 100 ng/ml recombinant human CXCL8 (rhIL-8; R&D Systems). After 0, 24, 48 and 96 h, we applied CellTiter^®^ 96 Aqueous One Solution Reagent (Promega, Madison, WI). The absorbance was measured by a microplate reader (Infinite 200 PRO; Tecan, Mannedorf, Switzerland) at 492 nm.

### Transwell migration assay and invasion assay

For the migration assay, TE cells (5.0 × 10^5^ cells/ well) in serum-free media were plated on the upper transwell inserts with an 8-μm pore filter (BD Falcon, Lincoln Park, NY) in 24-well plates. For the invasion assay, TE cells (5.0 × 10^5^ cells/ well) in serum-free media were plated on the inserts of a Corning^®^ BioCoat™ Matrigel^®^ Invasion Chamber (Corning, Tewksbury, MA) in 24-well plates. Medium containing 1% FBS was added in the lower chamber. Recombinant human CXCL8, inhibitors, or neutralizing antibodies were added in the upper chamber and incubated at 37°C. After 24 h or 48 h, the cells were stained with Diff-Quik (Sysmex, Kobe, Japan), and then the cells on the upper surface of the membrane were removed with a cotton swab and air-dried. Five images at 100× magnification were obtained from each membrane with a CCD camera, and the number of cells was counted. The inhibitors against PI3K (LY294002) and MEK1/2 (PD98059) were purchased from Cell SignaIing Technology. The neutralizing antibodies were as follows (all from Abcam): normal mouse IgG (#ab188776); mouse antibody against CXCR1 (#ab10400); mouse antibody against CXCR2 (#ab10401); mouse antibody against CXCL8 (#ab18672).

### Co-culture transwell migration assay and invasion assay

PBMos (1.0 × 10^5^ cells/well) were seeded on the lower chamber in 24-well plates with M-CSF (25 ng/ml; R&D Systems) for 6 days to induce macrophages, and then incubated with 50% TECMs to induce TAM-like macrophages. After 2 days, the media were replaced with serum-free media. TE cells (5.0 × 10^5^ cells/well) in serum-free media plated on the upper transwell inserts. Transwell migration and invasion assays were performed as described above.

### *CXCR1* and *CXCR2* knockdown by small interfering RNA

Cancer cell lines were transfected with 20 nM small interfering RNA (siRNA) targeting human *CXCR1 (IL-8RA* siRNA, sc-40026, Santa Cruz) and *CXCR2 (IL-8RB* siRNA, sc-40028, Santa Cruz) using Lipofectamine® RNAiMAX (Invitrogen). Control siRNA (Sigma-Aldrich) was used as the negative control.

### Enzyme-linked immunosorbent assay (ELISA)

PBMo-derived macrophages, TAM-like macrophages and ESCC cell lines were cultured in 24-well plates (1 × 10^5^ cells/well) with RPMI-1640 10% FBS and 1% antibiotic-antimycotic. After 48 h, the CXCL8 levels in the cell culture supernatants were determined by the Quantikine ELISA Human IL-8 immunoassay (R&D Systems) according to the manufacturer's instructions. The optical density of each well was determined by the Infinite 200 PRO microplate reader at 492 nm. The CXCL8 concentration of each sample was calculated using a standard curve and the measured absorbance.

### Immunohistochemistry

Antigen retrieval of 10% formalin-fixed and paraffin-embedded tissues was heat-induced in citrate buffer, pH 6.0. Immunohistochemistry was performed using EnVision Dual Link System-HRP, 3.3′-diaminobenzidine (Dako Cytomation, Glostrup, Denmark). The following antibodies were used to detect cellular antigens: mouse antibody against CXCL8 (1:20, #ab18672, Abcam), mouse antibody against CXCR1 (1:50, #ab10400, Abcam), mouse antibody against CXCR2 (1:20, #22106, LifeSpan BioSciences, Seattle, WA). We evaluated the immunohistochemical staining intensity of the cancer nests as a qualitative score compared to that of corresponding normal esophageal epithelium: CXCL8, negative, low, and high; CXCR1, low and high; CXCR2, low and high.

### Statistical analysis

All experiments were performed in triplicate and independently conducted three times. The results are expressed as mean ± SEM, and statistical significance was analyzed by two-sided Student's *t*-test or one-way ANOVA. The relationships between clinicopathological factors and immunohistochemical results were estimated by χ^2^ test. Disease-free and overall survival curves were estimated by the Kaplan-Meier method compared by log-rank test. The significance of parameters in a multivariate analysis was tested using the Cox proportional hazard regression model. A *p*-value < 0.05 was considered significant. Statistical analyses were carried out using SPSS Statistics ver. 22 (IBM, Chicago, IL).

## SUPPLEMENTARY FIGURES



## Abbreviations

Ct: threshold cycle
CXCL8: C-X-C chemokine ligand 8
DFS: disease-free survival
DMEM: Dulbecco's Modified Eagle's Medium
ESCC: esophageal squamous cell carcinoma
HRP: horseradish peroxidase
IL-4: interleukin 4
IL-8: interleukin 8
M-CSF: macrophage-colony stimulating factor
OS: overall survival
PBMC: peripheral blood mononuclear cell
PBMo: peripheral blood monocyte
TECM: conditioned medium of the TE series ESCC cell line
