# Supplementary material for: CXCL8 derived from tumor-associated macrophages and esophageal squamous cell carcinomas contributes to tumor progression by promoting migration and invasion of cancer cells
Source: Oncotarget. 2017 Nov 20;8(62):106071–88. doi: 10.18632/oncotarget.22526 (PMC5739702; doi:10.18632/oncotarget.22526)
Supplement: Supplementary file 1 [file oncotarget-08-106071-s001.pdf]

CXCL8 derived from tumor-associated macrophages and esophageal squamous cell carcinomas contributes to tumor progression by promoting migration and invasion of cancer cells

SUPPLEMENTARY MATERIALS

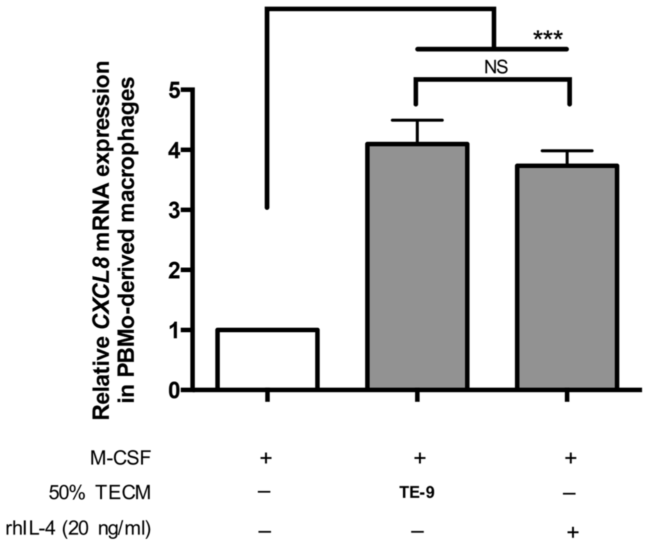

Supplementary Figure 1: rhIL-4 up-regulated the expression level of CXCL8 in PBMo-derived macrophages. The mRNA level of *CXCL8* in PBMo-derived macrophages stimulated with 50% TE-9 CM or rhIL-4 (20 ng/ml) was determined by quantitative RT-PCR. The data were normalized to *GAPDH* as an internal control. Data are mean ± SEM in triplicate. $^{***}p < 0.001$.

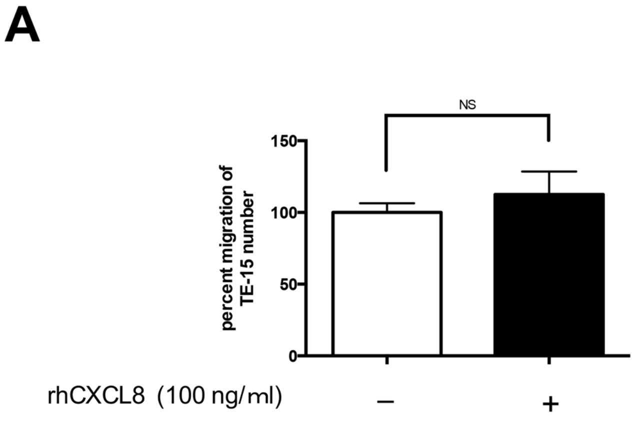

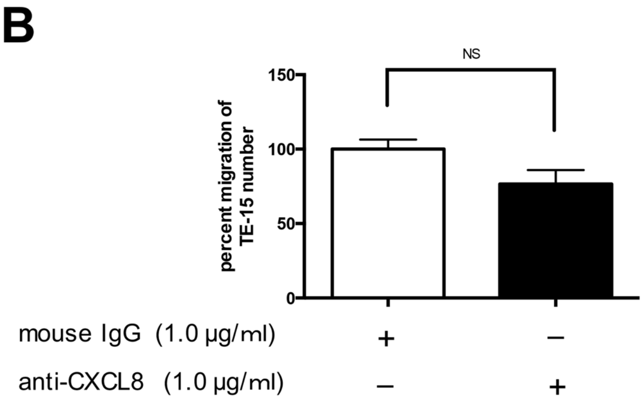

Supplementary Figure 2: rhCXCL8 did not induce migration of TE-15. Neutralizing antibody against CXCL8 tended to suppress migration of TE-15. **(A)** For the transwell migration assay, TE-15 cells were plated on the transwell in serum-free RPMI-1640 at 1.0×10^6 cells/well. rhCXCL8 was added in the upper chamber at 100 ng/ml. The cell inserts were set on 24-well plates in RPMI-1640 with 10% FBS for 48 h. The migrated cells on the underside of the membrane were stained by Diff-Quik and counted. **(B)** TE-15 cells were plated on the transwell in serum-free RPMI-1640 at 1.0×10^6 cells/well. Neutralizing antibody against CXCL8 was added in the upper chamber at 1.0 μg/ml. The cell inserts were set on 24-well plates in RPMI-1640 with 10% FBS for 48 h. The migrated cells on the underside of the membrane were stained by Diff-Quik and counted. The results are mean ± SEM. NS, not significant.

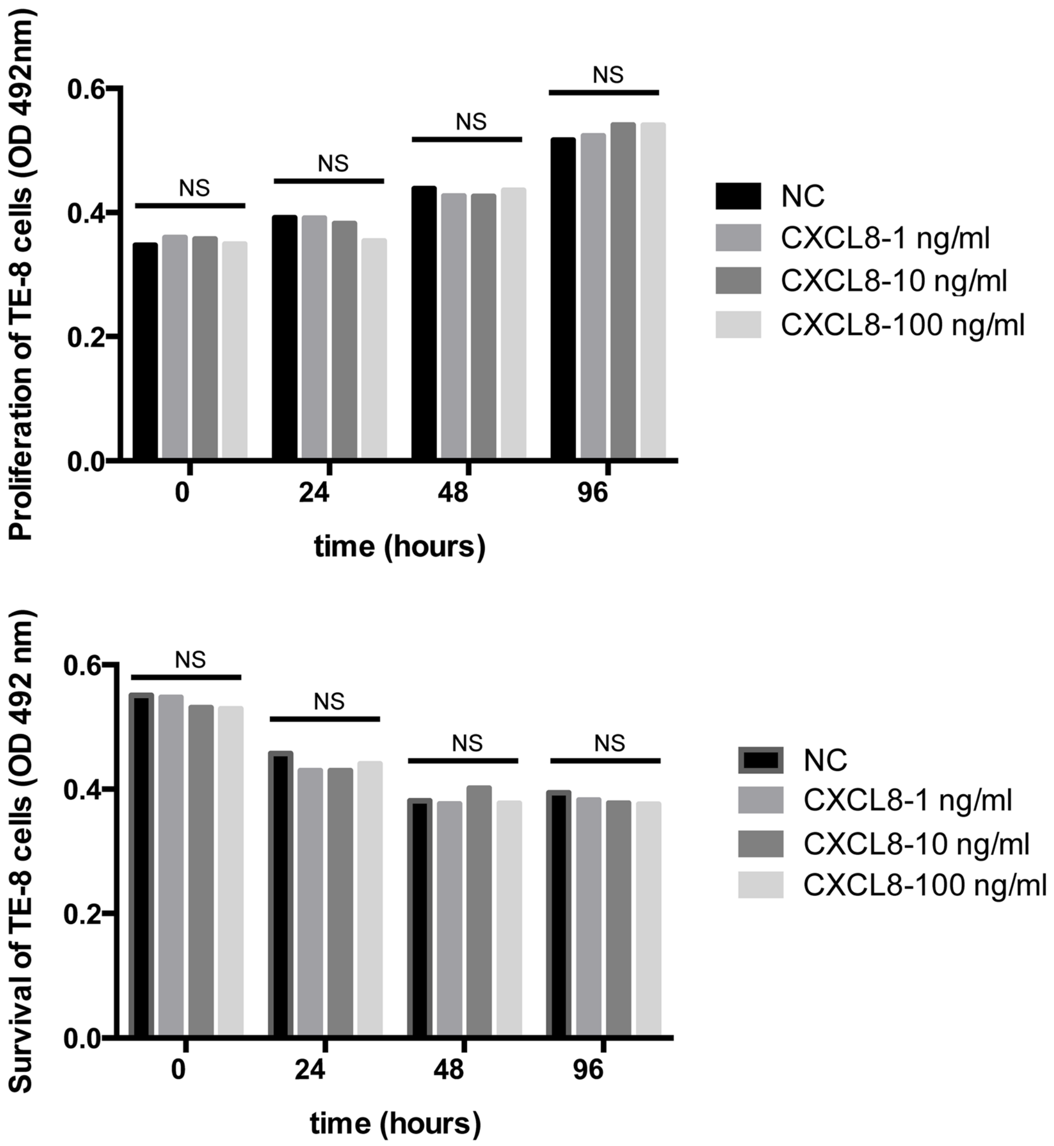

Supplementary Figure 3: CXCL8 did not promote growth and survival of TE-8 cells. TE-8 seeded on 96 well plate with serum free medium or medium with 1% serum treated with CXCL8 (0, 1, 10, 100 ng/ml) for 96 h. Cell growth and survival activity were assessed by MTS assay. The results are presented as the mean ± SEM. NC, negative control; NS, not significant.

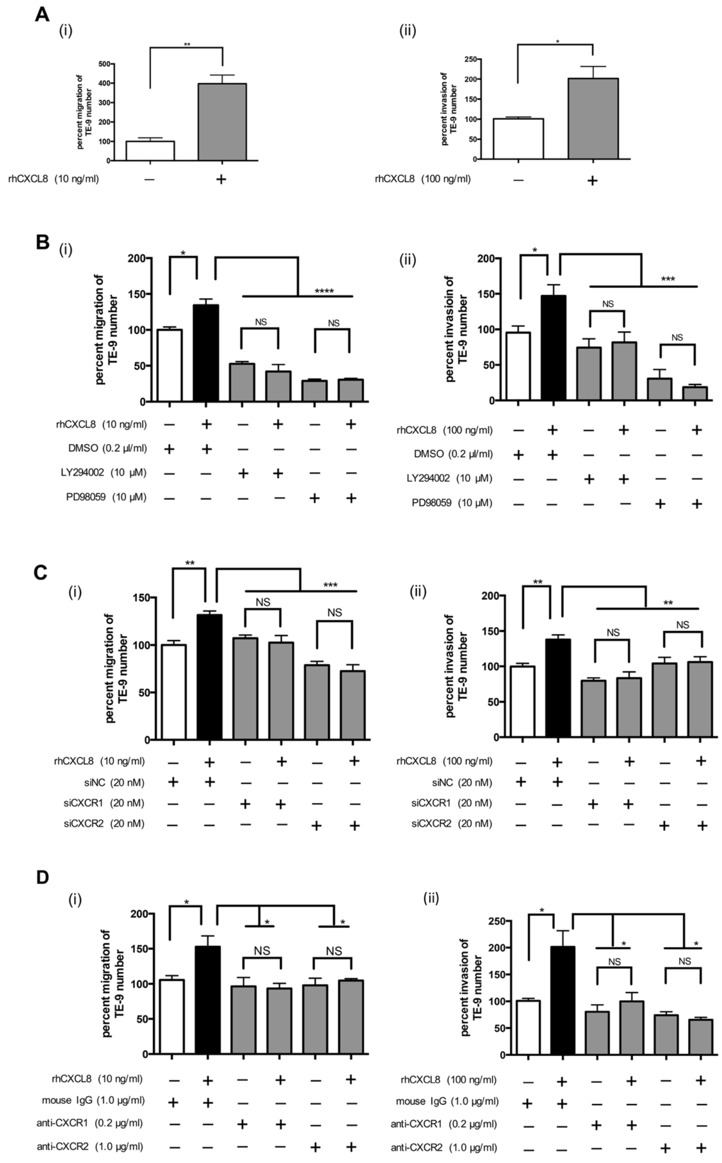

Supplementary Figure 4: CXCL8 promoted the migration and invasion of TE-9 cells. (A) (i) For the transwell migration assay, TE-9 cells were plated on the transwell in serum-free RPMI-1640 at 5.0×10^5 cells/well. rhCXCL8 was added in the upper chamber at 10 ng/ml. The cell inserts were set on 24-well plates in RPMI-1640 with 1% FBS for 24 h. The migrated cells on the underside of the membrane were stained by Diff-Quik and counted. The results are mean ± SEM. (ii) For the transwell invasion assay, TE-9 cells were seeded on a transwell coated with matrigel in serum-free RPMI-1640 at 5.0×10^5 cells/well. rhCXCL8 was added in the transwell at 100 ng/ml. The cell inserts were set on 24-well plates in RPMI-1640 with 1% FBS for 48 h. The invaded cells on the underside of the membrane were stained by Diff-Quik and counted. **(B)** (i) TE-9 cells were plated on the upper chamber with rhCXCL8 at 10 ng/ml combined with the inhibitor against PI3K (LY294002, 10 μM) or MEK1/2 (PD98059, 10 μM). DMSO (0.2μl/ml) was added as negative control. After 24 h, the migrated cells were counted. (ii) TE-9 cells were plated on the upper transwell coated with matrigel. rhCXCL8 was added in the transwell combined with LY294002 (10 μM) or PD98059 (10 μM). DMSO (0.2μl/ml) was added to negative control. After 48 h, the invaded cells were counted. **(C)** (i) *CXCR1*- or *CXCR2*-silenced TE-9 cells were plated on the upper transwell with rhCXCL8 at 10 ng/ml. After 24 h, the migrated cells were counted. (ii) *CXCR1*- or *CXCR2*-silenced TE-9 cells were plated on the upper transwell coated with matrigel. rhCXCL8 was added to the transwell at 100 ng/ml. After 48 h, the invaded cells were counted. **(D)** (i) TE-9 cells were plated on the upper transwell with rhCXCL8 at 10 ng/ml combined with the neutralizing antibody against CXCR1 (0.2 μg/ml) or CXCR2 (1.0 μg/ml). Mouse IgG (1.0 μg/ml) was added to negative control. The concentrations of neutralizing antibodies were based on the manufacturer's instructions. (ii) TE-9 cells were plated on the upper transwell coated with matrigel. rhXCL8 was added to the upper transwell at 100 ng/ml combined with the neutralizing antibody against CXCR1 (0.2 μg/ml) or CXCR2 (1.0 μg/ml). Mouse IgG (1.0 μg/ml) was added to negative control. After 48 h, the invaded cells were counted. NS, not significant; $^{*}p < 0.05$, $^{**}p < 0.01$, $^{***}p < 0.001$, $^{****}p < 0.0001$.

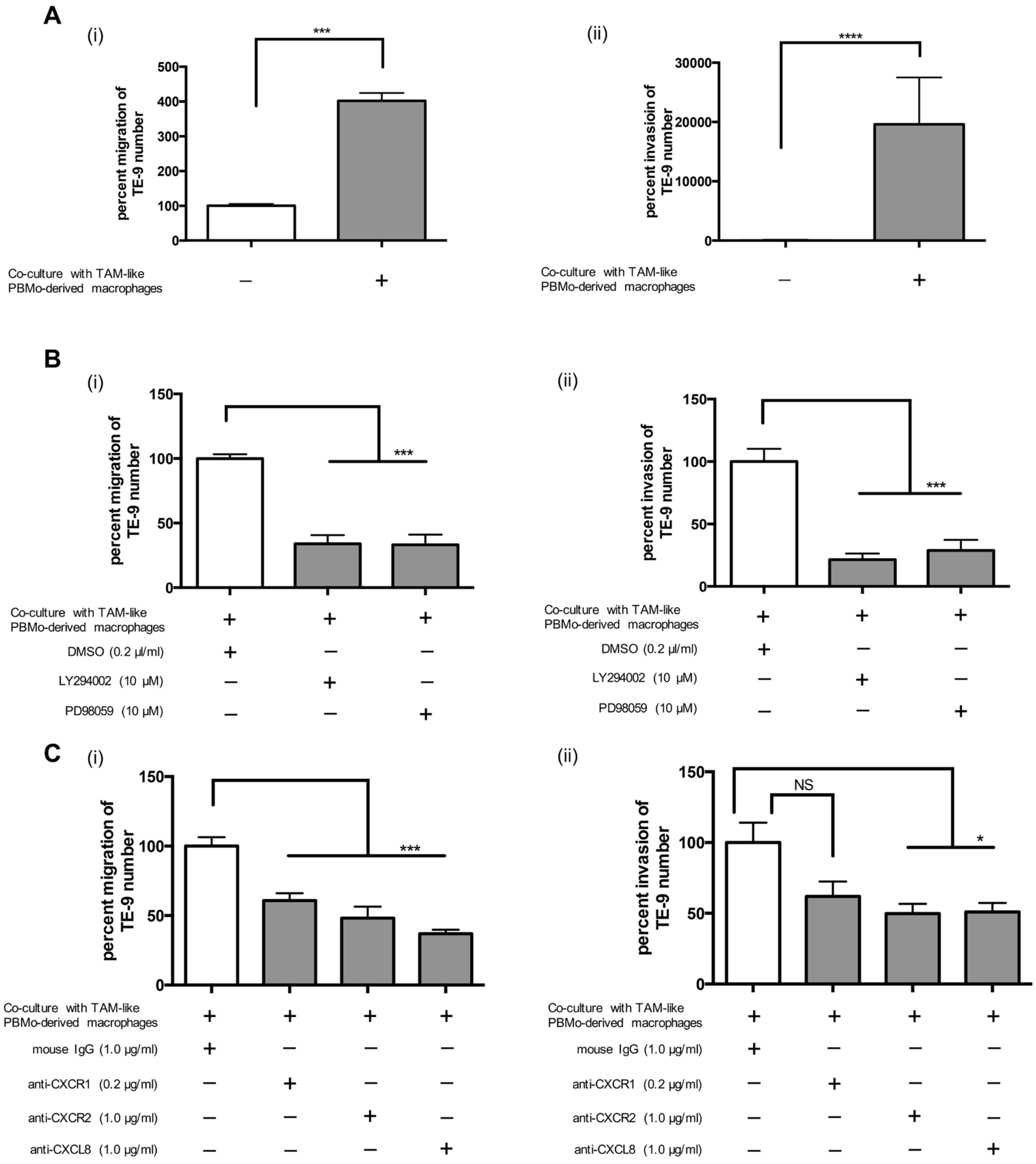

Supplementary Figure 5: TAM-like PBMo derived macrophages induced the migration and invasion of TE-9 cells. **(A)** PBMos (1.0×10^5 cells/well) were seeded on the lower chamber of 24-well plates with M-CSF (25 ng/ml) for 6 days to induce PBMo-derived macrophages, then incubated with 50% TE-9 CM to induce TAM-like PBMo-derived macrophages. After 2 days, the media were replaced with serum-free media. (i) TE-9 cells were plated on the upper transwell at 5.0×10^5 cells/well and set on the plate. After 24 h, the migrated cells were counted. (ii) TE-9 cells were plated on the upper transwell coated with matrigel at 5.0×10^5 cells/well and set on the plate. After 48 h, the invaded cells were counted. **(B)** (i) TE-9 cells were plated on the upper transwell with the inhibitor against PI3K (LY294002, 10 nM) or MEK1/2 (PD98059, 10 nM). DMSO (0.2µl/ml) was added to negative control. After 24 h, the migrated cells were counted. (ii) TE-9 cells were plated on the upper matrigel-coated transwell with LY294002 (10 nM) or PD98059 (10 nM). After 48 h, the invaded cells were counted. **(C)** (i) TE-9 cells were plated on the upper transwell with the neutralizing antibodies against CXCR1 (0.2 µg/ml), CXCR2 (1.0 µg/ml) or CXCL8 (1.0 µg/ml). Mouse IgG (1.0 µg/ml) was added to negative control. After 24 h, the migrated cells were counted. The concentrations of neutralizing antibody were based on manufacturer's instructions. (ii) TE-9 cells were plated on the upper matrigel-coated transwell with the neutralizing antibodies against CXCR1 (0.2 µg/ml), CXCR2 (1.0 µg/ml) or CXCL8 (1.0 µg/ml). Mouse IgG (1.0 µg/ml) was added to negative control. After 48 h, the invaded cells were counted. The results are mean ± SEM. $^{*}p < 0.05$, $^{**}p < 0.01$, $^{***}p < 0.001$, $^{****}p < 0.0001$.

A

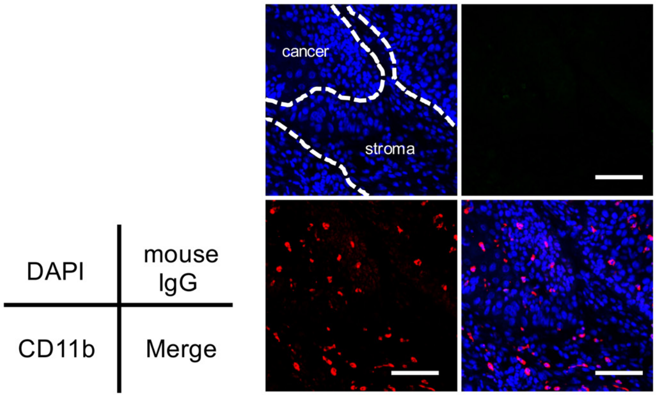

B

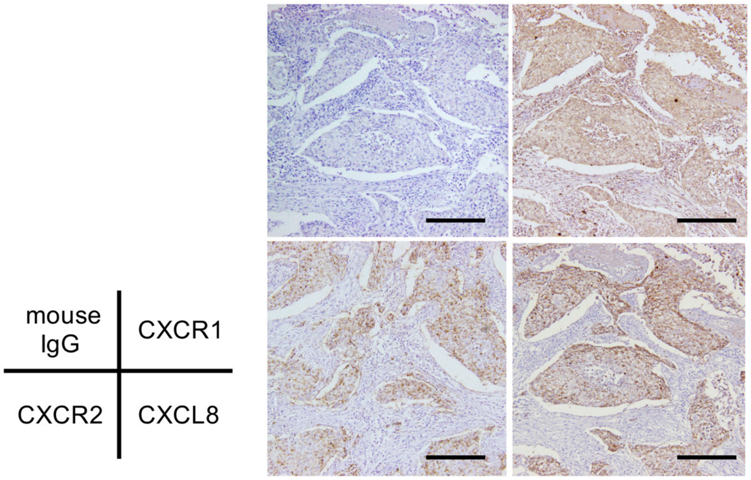

Supplementary Figure 6: Negative control study of imuunofluorescene and immunohistochemistry. **(A)** A double immunofluorescence analysis was performed using normal mouse IgG (negative control) (green) in the replacement of mouse anti-CXCL8 antibody and anti-CD11b (red) antibodies on human ESCC tissue. Nuclei were stained with DAPI (blue). Scale bar, 100 µm. **(B)** Immunohistochemical staining using the same human ESCC tissue. Mouse IgG was used as negative control. Scale bar, 100 µm.

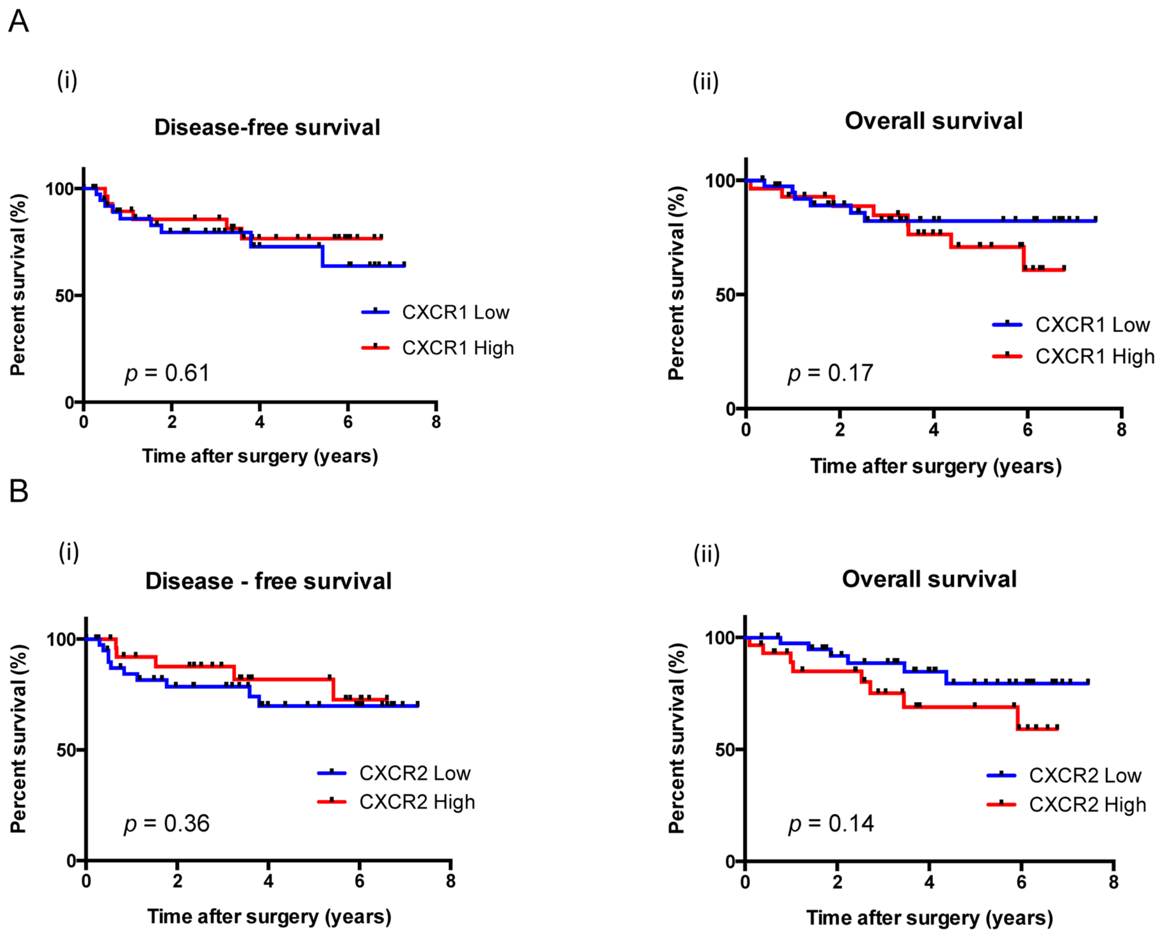

Supplementary Figure 7: Expression of CXCR1 and CXCR2 did not associated with disease-free survival and overall survival. (A) (i) (ii) Expression levels of CXCR1 were not related to disease-free survival and overall survival. **(B)** (i) (ii) Expression levels of CXCR2 were not related to disease-free survival and overall survival.
